# miR-21 improves the neurological outcome after traumatic brain injury in rats

**DOI:** 10.1038/srep06718

**Published:** 2014-10-24

**Authors:** Xin-Tong Ge, Ping Lei, Hai-Chen Wang, An-Ling Zhang, Zhao-Li Han, Xin Chen, Sheng-Hui Li, Rong-Cai Jiang, Chun-Sheng Kang, Jian-Ning Zhang

**Affiliations:** 1 Laboratory of Neuro-Trauma, Tianjin Neurological Institute, Tianjin, China; 2 Key Laboratory of Post-trauma Neuro-repair and Regeneration in Central Nervous System, Ministry of Education, Tianjin, China; Tianjin Key Laboratory of Injuries, Variations and Regeneration of Nervous System, Tianjin, Tianjin, China; 3 Department of Neurosurgery, Tianjin Medical University General Hospital, Tianjin, China; 4 Department of Geriatrics, Tianjin Medical University General Hospital, Tianjin, China; Laboratory of Neuro-Trauma and Neurodegenerative Disorders, Tianjin Geriatrics Institute, Tianjin, China; 5 Department of Neurology, Duke University School of Medicine, Durham, North Carolina, U.S.A.; 6 Laboratory of Neuro-Oncology, Tianjin Neurological Institute, Tianjin, China

## Abstract

The expression levels of microRNAs (miRNAs) including miR-21, have been reported to change in response to traumatic brain injury (TBI), suggesting that they may influence the pathophysiological process in brain injury. To analyze the potential effect of miR-21 on neurological function after TBI, we employed the fluid percussion injury rat model and manipulated the expression level of miR-21 in brain using intracerebroventricular infusion of miR-21 agomir or antagomir. We found that upregulation of miR-21 level in brain conferred a better neurological outcome after TBI by improving long-term neurological function, alleviating brain edema and decreasing lesion volume. To further investigate the mechanism underlying this protective effect, we evaluated the impact of miR-21 on apoptosis and angiogenesis in brain after TBI. We found that miR-21 inhibited apoptosis and promoted angiogenesis through regulating the expression of apoptosis- and angiogenesis-related molecules. In addition, the expression of PTEN, a miR-21 target gene, was inhibited and Akt signaling was activated in the procedure. Taken together, these data indicate that miR-21 could be a potential therapeutic target for interventions after TBI.

Traumatic brain injury (TBI) is one of the leading causes of injury induced mortality and disability, especially in children and young adults. It is estimated that 10 million people are affected by TBI world-wide annually, and TBI will become the third most common cause of global disease burden by 2020^1^. TBI leads to a complex series of pathological events after the primary insult including cellular apoptosis, impaired local blood supply and blood-brain barrier (BBB) disruption, which is known as secondary brain damage^2^,^3^. Secondary brain damage is the major cause of brain edema, intracranial hypertension and subsequent neurological dysfunction^4^. Since the primary insult cannot be influenced therapeutically, reducing secondary brain damage by alleviating cellular apoptosis and promoting angiogenesis can significantly improve the prognosis of TBI.

MicroRNAs (miRNAs), a novel family of non-protein coding short RNA molecules that negatively modulate protein expression, have been implicated in the etiology of a variety of diseases including cancer, cardiovascular diseases, diabetes mellitus and neurological diseases^5^. However, little attention has been paid to their roles in acute diseases, such as TBI. Recent studies both *in vivo*^6^ and *in* *vitro*^7^ have shown that the levels of temperature-sensitive miRNAs (miR-34a, miR-874 and miR-451) change in TBI after hypothermia therapy and are involved in the regulation of cellular apoptosis after injury. It has been reported that miR-34a can regulate the proliferation of neural stem cells in the hippocampus through crosstalk with Notch signaling^8^, and miR-107 can influence the process of wound repair by regulating granulin expression after TBI^9^. In addition, several studies examined the miRNA profiles of cerebral spinal fluid and blood samples from patients and TBI rats, and identified potential miRNAs biomarkers in TBI^10^,^11^.

The role of miR-21 in TBI is not well documented. Redell et al.^12^,^13^ and our group^14^ have identified that miRNA expression levels change in the hippocampus and injured cerebral cortex of TBI rats. This was demonstrated by profiling miRNAs and qRT-PCR, which showed an increase in the expression level of miR-21 in both brain regions from 6 h to 72 h post-injury. Several target genes such as PDCD4 and Tiam1 were predicted to be regulated by miR-21 after TBI^13^. These findings suggested that miR-21 is a potential biomarker and therapeutic target in TBI. To explore the function and mechanism of miR-21 in TBI, we focused on studying its effects on neurological outcome and related pathological changes including cellular apoptosis and angiogenesis. The results will potentially open a new avenue of therapeutic strategies for TBI by manipulating miRNA levels.

## Results

### Altered miR-21 expression in the traumatic foci after TBI and intracerebroventricular infusion of miR-21 oligomers

**Figure 1:**
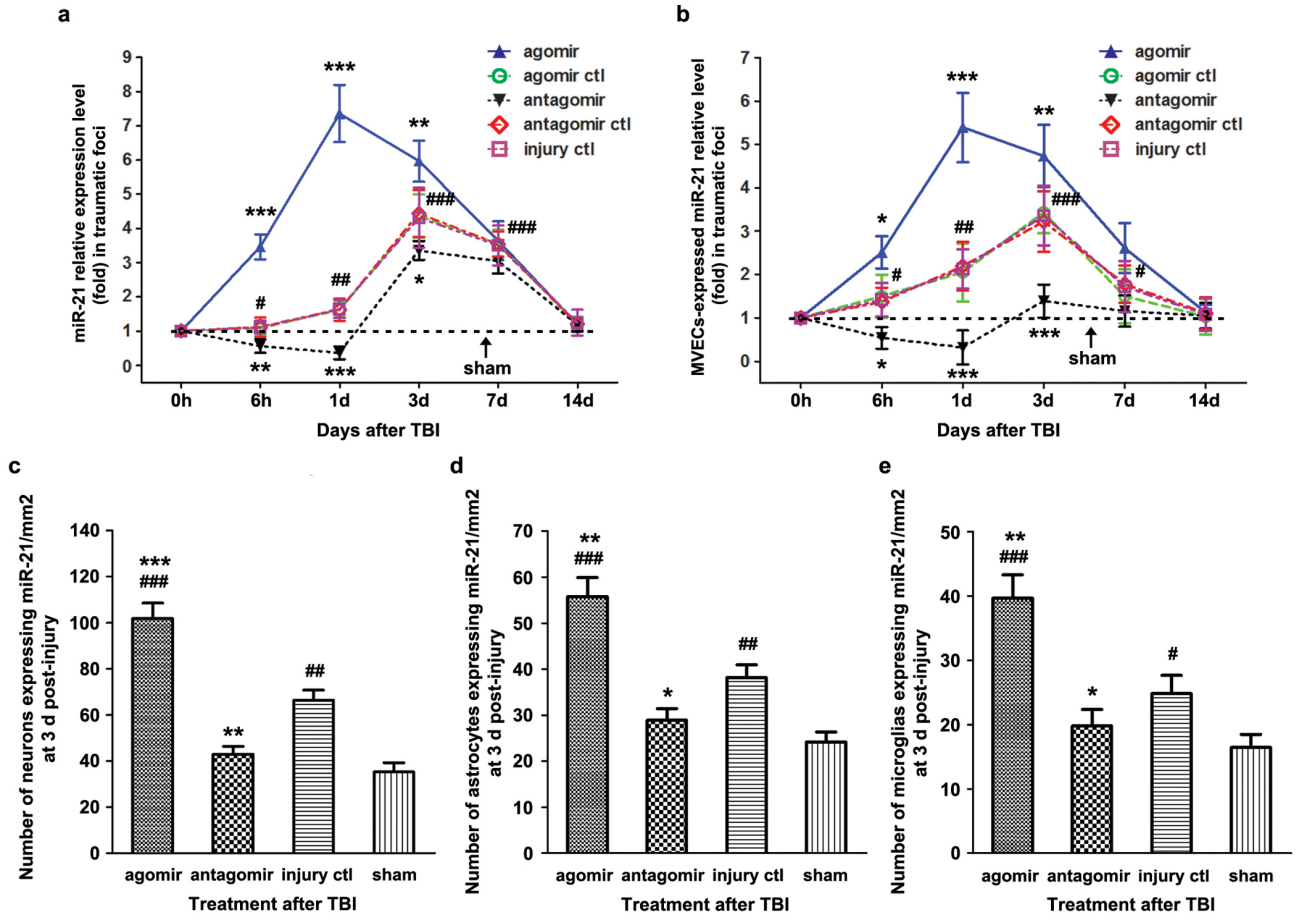
Altered miR-21 level in the traumatic foci after TBI and intervention with miR-21 oligomers. The temporal profile (from 0 h to 14 d post-injury) of miR-21 level (a) and microvascular endothelial cells (MVECs) – expressed miR-21 level (b) in the traumatic foci determined by qRT-PCR. The quantitative data of a and b were analyzed using the 2^−ΔΔCt^ method, in which the miR-21 levels of the *sham* group (presented as the dotted line) were used as controls. The quantitative data of miR-21 immunopositive neurons (c), astrocytes (d) and microglias (e) at 3 d post-injury detected by combined miRNA in-situ hybridization and immunofluorescence staining. The data are expressed as mean ± SD. (n = 6) (*P < 0.05, **P < 0.01, ***P < 0.001 versus the *injury ctl* group. ^#^P < 0.05, ^##^P < 0.01, ^###^P < 0.001 versus the *sham* group. Specifically, the pound signs in 1a represent the *injury ctl* group versus the *sham* group.)

We detected miR-21 expression levels in the traumatic foci, which were defined as the impacted area with a diameter of 7 mm including injured cerebral cortex and ipsilateral hippocampus, at different time-points from 0 h to 14 d post-injury using qRT-PCR (Figure 1a). The expression of miR-21 in the *injury ctl* group was increased at 6 h post-injury, reached the peak level at 3 d post-injury and then gradually declined to baseline at 14 d post-injury. Compared with the *injury ctl* group, the miR-21 expression level was significantly increased (or decreased) in the *agomir* (or *antagomir*) group at 6 h, 1 d and 3 d post-injury. These data indicated that TBI leads to upregulation of miR-21 in the traumatic foci and its expression can be manipulated (upregulated or downregulated) by intracerebroventricular infusion of miR-21 agomir or antagomir. The temporal profile of miR-21 level in the contralateral cerebral cortex after TBI is shown in supplementary Figure 1.

To investigate the in-situ expression of miR-21 in microvascular endothelial cells (MVECs), we isolated MVECs from the traumatic foci and quantified the temporal profile of miR-21 level using qRT-PCR (Figure 1b). We found that MVECs-expressed miR-21 in the *injury ctl* group was increased at 6 h post-injury, reached the peak level at 3 d post-injury and then gradually declined to baseline at 14 d post-injury. Compared with the *injury ctl* group, the miR-21 level in MVECs was increased (or decreased) at 6 h, 1 d and 3 d post-injury after miR-21 agomir (or antagomir) infusion.

**Figure 2:**
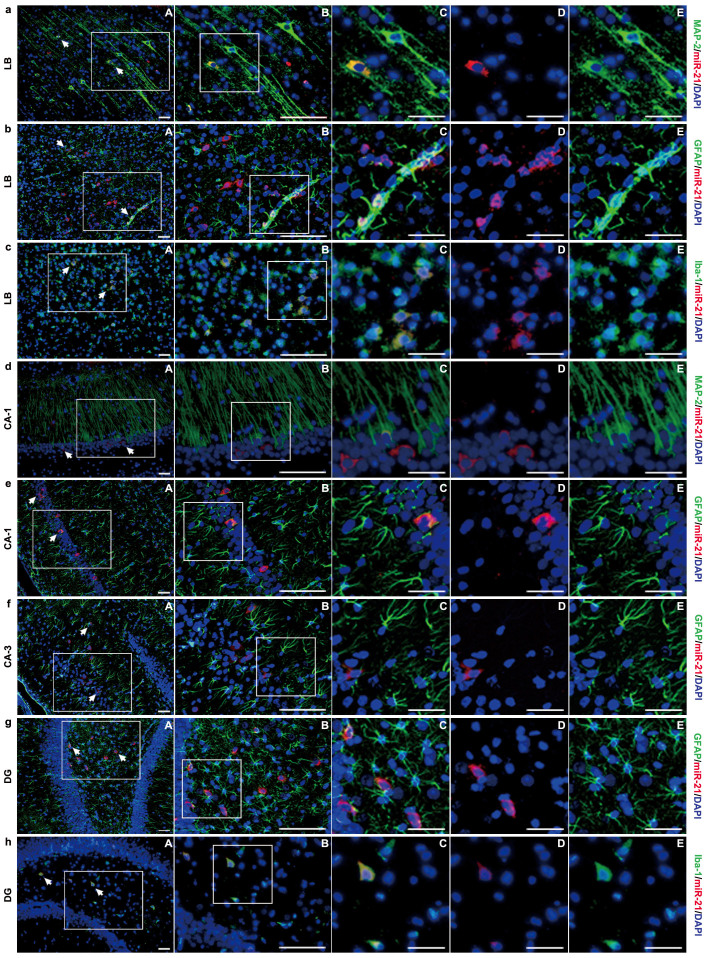
Representative images of the immunostaining of in-situ miR-21 expressed by neurons, astrocytes and microglias in different areas of brain. The combined staining of miR-21/MAP-2 (a), miR-21/GFAP (b), miR-21/Iba-1 (c) lesioned boundary (LB, scope see Figure 3e) of cerebral cortex. The combined staining of miR-21/MAP-2 (d) and miR-21/GFAP (e) in the CA1 (a subdivision of Ammon's horn). The combined staining of miR-21/GFAP (f) in the CA3 (a subdivision of Ammon's horn). The combined staining of miR-21/GFAP (g) and miR-21/Iba-1 (h) in the dentate gyrus (DG). In each set of the figures, the immunostained areas in the white box were magnified from Figure A to B and C. A group of figures (Figure C, D, E) in the same location were presented to better illustrate the effect of combined immunostaining. All figures were captured from the *sham* group. Scale bars: A–B, 50 μm; C–E, 20 μm. (n = 6).

In addition, we measured the in-situ expression of miR-21 in various cells of the central nervous system (CNS) including neurons, astrocytes and microglias at 72 h post-injury using combined miRNA in-situ hybridization (ISH) and immunofluorescence (IF) staining (Figure 1c–1e, Figure 2, Supplementary Figure 2). The in-situ staining using control probe is shown in Supplementary Figure 3. The results indicated that the expression levels of miR-21 in these cells were all increased at 3 d post-injury, and were significantly upregulated (or downregulated) after infusing miR-21 agomir (or antagomir). These data demonstrated that intracerebroventricularly infused miR-21 agomir or antagomir can be taken up by different types of brain cells, so that regulate miR-21 level in these cells.

### Upregulation of miR-21 level in brain improved the neurological outcome after TBI

The impacts of miR-21 on the neurological outcome of TBI rats were evaluated from three aspects: the long-term neurological function, represented by spatial learning ability and reference memory; the severity of brain edema after acute trauma, which is associated with severe clinical complications such as progressive intracranial hypertension and brain hernia that related to the neurological outcome^15^; and the lesion volume of the traumatic hemisphere, which is a surrogate marker for the histopathological outcome that impacts the neurological outcome.

**Figure 3:**
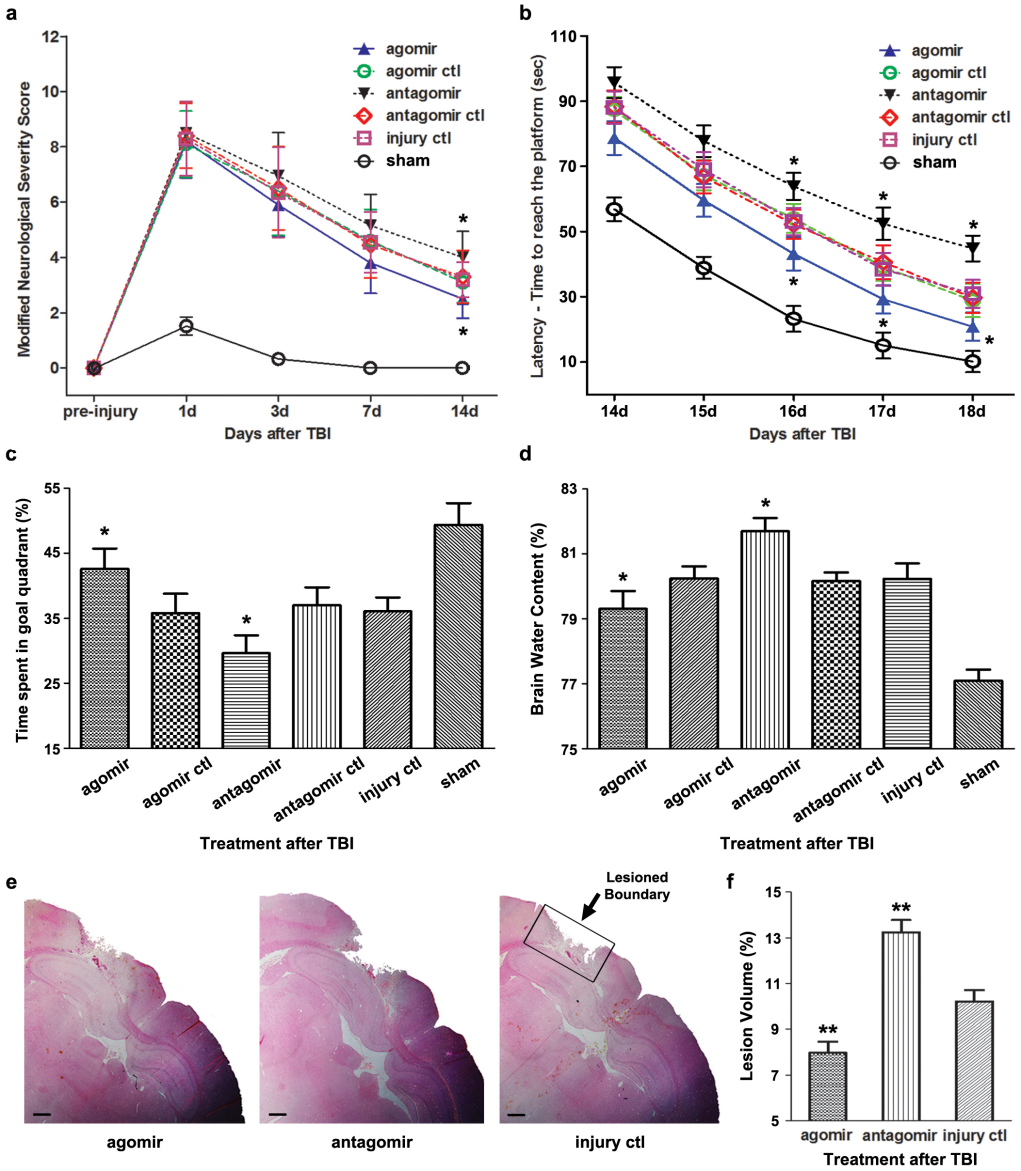
The impact of regulating brain miR-21 level on the long-term neurological function, brain edema and histopathological outcomes of TBI rats. The long-term neurological function evaluated by the mNSS test (a) (n = 10) and Morris Water Mass test (n = 6), which includes the spatial acquisition trial (b) and the probe trial (c). The quantitative data of brain water content measurement for lesioned cerebral hemisphere and cerebellum at 72 h post-injury (d) (n = 6). The H&E staining (e) and the quantitative data of lesion volume (f) at 14 d post-injury (n = 10). The data are expressed as mean ± SD (a, c, d, f) or mean ± SEM (b). Scale bars in 3e: 100 μm. (*P < 0.05, **P < 0.01 versus the *injury ctl* group).

The modified Neurological Severity Score (mNSS) test and the Morris Water Mass (MWM) test were performed to evaluate the long-term neurological function. For the mNSS test, as shown in Figure 3a, there was no difference in the neurological score among all experimental groups at 24 h post-injury, indicating that rats in different groups had relatively comparable injuries (one-way ANOVA, F(4,45) = 0.167, P = 0.954). The recovery of neurological function began at 3 d and lasted to 14 d post-injury, when rats suffered from residual neurological deficiencies with high scores on sensory and beam balance tests. No difference was observed from 1 d to 7 d post-injury. At 14 d post-injury, the neurological score of the *agomir* group was decreased compared with the *injury ctl* group. Meanwhile, an increased neurological score was observed in the *antagomir* group. In the MWM test, the spatial acquisition trial was conducted to test spatial learning ability. Escape latency, which represents the capability to navigate from a start location to a submerged platform, gradually decreased from 14 d to 18 d post-injury, indicating that a spatial memory was established (repeated-measures ANOVA, F(4,120) = 775.2, P < 0.001). Furthermore, compared with the *injury ctl* group, escape latency was improved in the *agomir* group and impaired in the *antagomir* group at 18 d post-injury (Figure 3b). The probe trial was performed to test the retrograde reference memory, where more time spent in the goal quadrant demonstrates better memory. Compared with the *injury ctl* group, the *agomir* group displayed an increase in the time spent in goal quadrant, whereas a decrease was observed in the *antagomir* group (Figure 3c). No difference was observed in swim speed among the groups (Supplementary Figure 4), indicating that the different performance was not due to motor impairments.

The severity of brain edema was evaluated by measuring brain water content at 72 h post-injury, when peak brain edema develops after TBI according to previously reported^16^. We found that compared with the *injury ctl* group, the water content of lesioned cerebral hemisphere was decreased in the *agomir* group and increased in the *antagomir* group (Figure 3d).

We quantified the lesion volume of injured brain at 14 d post-injury. A significant decrease (or increase) in the lesion volume was observed in the *agomir* (or *antagomir*) group compared with the *injury ctl* group (Figure 3e, 3f). In addition, the lesion volume was correlated with the mNSS at 14 d post-injury (Spearman's rank correlation coefficient test, r = 0.434, P < 0.05), indicating that the histopathological outcome was significantly related to the neurological outcome.

Taken together, these results indicated that upregulation of miR-21 level in brain can improve the long-term neurological function, alleviate brain edema and decrease lesion volume. Thus, miR-21 upregulation was beneficial for improving neurological function after TBI.

### miR-21 attenuated cellular apoptosis and regulated the expression of apoptosis-related molecules in brain after TBI

**Figure 4:**
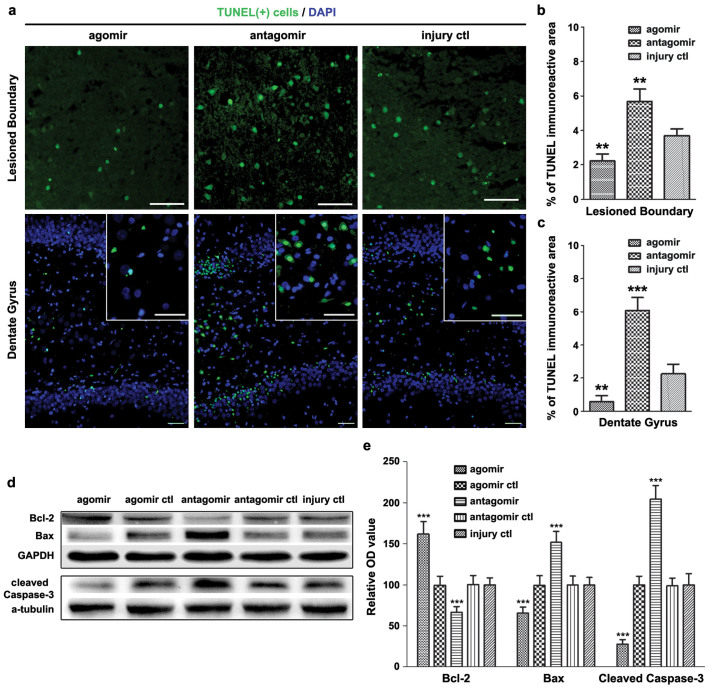
The impact of miR-21 on cellular apoptosis in brain after TBI. The immunostaining of apoptotic cells in the lesioned boundary (LB) of cerebral cortex and the dentate gyrus of ipsilateral hippocampus (DG) at 72 h post-injury (a). The quantitative data of immunostained apoptotic cells in a (b, c). The immunoblotting (d) and quantitative data (e) of apoptosis-related molecules (Bcl-2, Bax and cleaved Caspase-3) acquired from the traumatic foci at 72 h post-injury. The data are expressed as mean ± SD. Scale bars: 50 μm. (n = 6) (**P < 0.01, ***P < 0.001 versus the *injury ctl* group).

The cellular apoptosis in brain following TBI was evaluated by the TUNEL assay at 72 h post-injury. This time-point was chosen because miR-21 expression was most obviously increased at this time-point after TBI without the intervention. Therefore, the impact of miR-21 on the cellular apoptosis after TBI at this time-point could potentially be remarkable. We observed that the proportion of immunoreactive (apoptotic positive) area in the lesioned boundary (LB, scope see Figure 3e) of the cerebral cortex and the dentate gyrus (DG) of ipsilateral hippocampus were decreased (or increased) in the *agomir* (or *antagomir*) group compared with the *injury ctl* group (Figure 4a–4c). This result indicated that TBI leads to cellular apoptosis in the cerebral cortex and ipsilateral hippocampus, and that upregulation of miR-21 in brain can alleviate cellular apoptosis in vulnerable cell population. The impact of miR-21 on cellular apoptosis in contralateral brain after TBI was also evaluated (supplementary Figure 5). We then detected the expression levels of Bcl-2, Bax and cleaved Caspase-3 in brain to clarify the impact of miR-21 on apoptosis after TBI. Compared with the *injury ctl* group, the Bcl-2 level was increased in the *agomir* group and decreased in the *antagomir* group, while reverse changes were observed in the expression level of Bax and Caspase-3 (Figure 4d, 4e). These data suggested that the anti-apoptotic function of miR-21 after TBI was associated with its regulation on the expression of apoptosis-related molecules.

### miR-21 promoted angiogenesis and regulated the expression of angiogenesis-related molecules in brain after TBI

**Figure 5:**
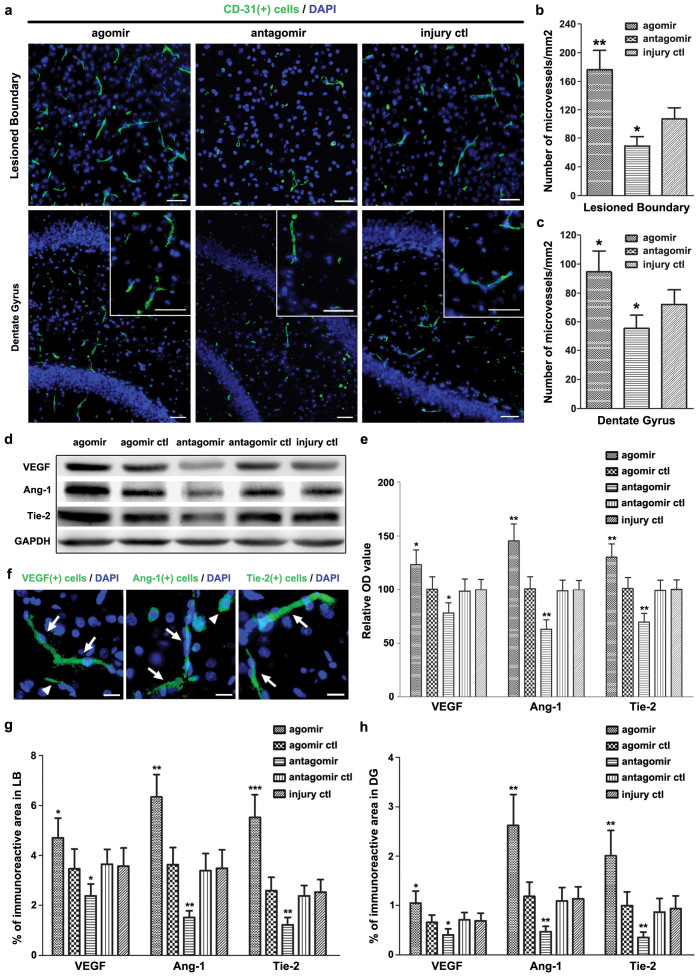
The impact of miR-21 on angiogenesis in brain after TBI. The immunostaining of microvessels marked by CD31 in the lesioned boundary (LB) of cerebral cortexand the dentate gyrus of ipsilateral hippocampus (DG) at 7 d post-injury (a). The quantitative data of immunostained microvessels in a (b, c). The immunoblotting of angiogenesis-related molecules (VEGF, Ang-1 and Tie-2) acquired from the traumatic foci at 7 d post-injury (d). The immunoreactive area located on the vessels (white arrow) and the extracellular space of endothelial cells (white triangle). The quantitative data of immunostained molecules in d (e). The representative images of immunostaining of VEGF, Ang-1 and Tie-2 in the LB of the *agomir* group (f). The quantitative data of immunostained VEGF, Ang-1 and Tie-2 in the LB (g) and the DG (h) at 7 d post-injury. The data are expressed as mean ± SD. Scale bars: 50 μm. (n = 6) (*P < 0.05, **P < 0.01, ***P < 0.001 versus the *injury ctl* group).

The restoration of local blood supply in brain after TBI was evaluated by measuring the microvascular density (MVD). We performed the experiments at 7 d post-injury because obvious changes in angiogenesis would be difficult to observe at an earlier time-point such as 3 d post-injury. We found that the MVD in the LB and the DG were increased (or decreased) in the *agomir* (or *antagomir*) group compared with the *injury ctl* group (Figure 5a–5c). Since the increase in the MVD could be induced by reduced microvessels death, we then detected the expression levels of angiogenesis-related molecules including VEGF, Angiopoietin-1 (Ang-1) and Tie-2 (receptor of Ang-1) in brain. Compared with the *injury ctl* group, the relative OD value (in the immunoblotting assay) of VEGF, Ang-1 and Tie-2, as well as their proportion of immunopositive areas (in the immunostaining assay) in the LB and the DG were all increased in the *agomir* group and decreased in the *antagomir* group (Figure 5d–5h). Taken together, these data suggested that upregulation of miR-21 level in brain can promote angiogenesis after TBI, which was associated with its regulation on the expression of angiogenesis-related molecules.

### Akt signaling was activated by miR-21 after TBI

**Figure 6:**
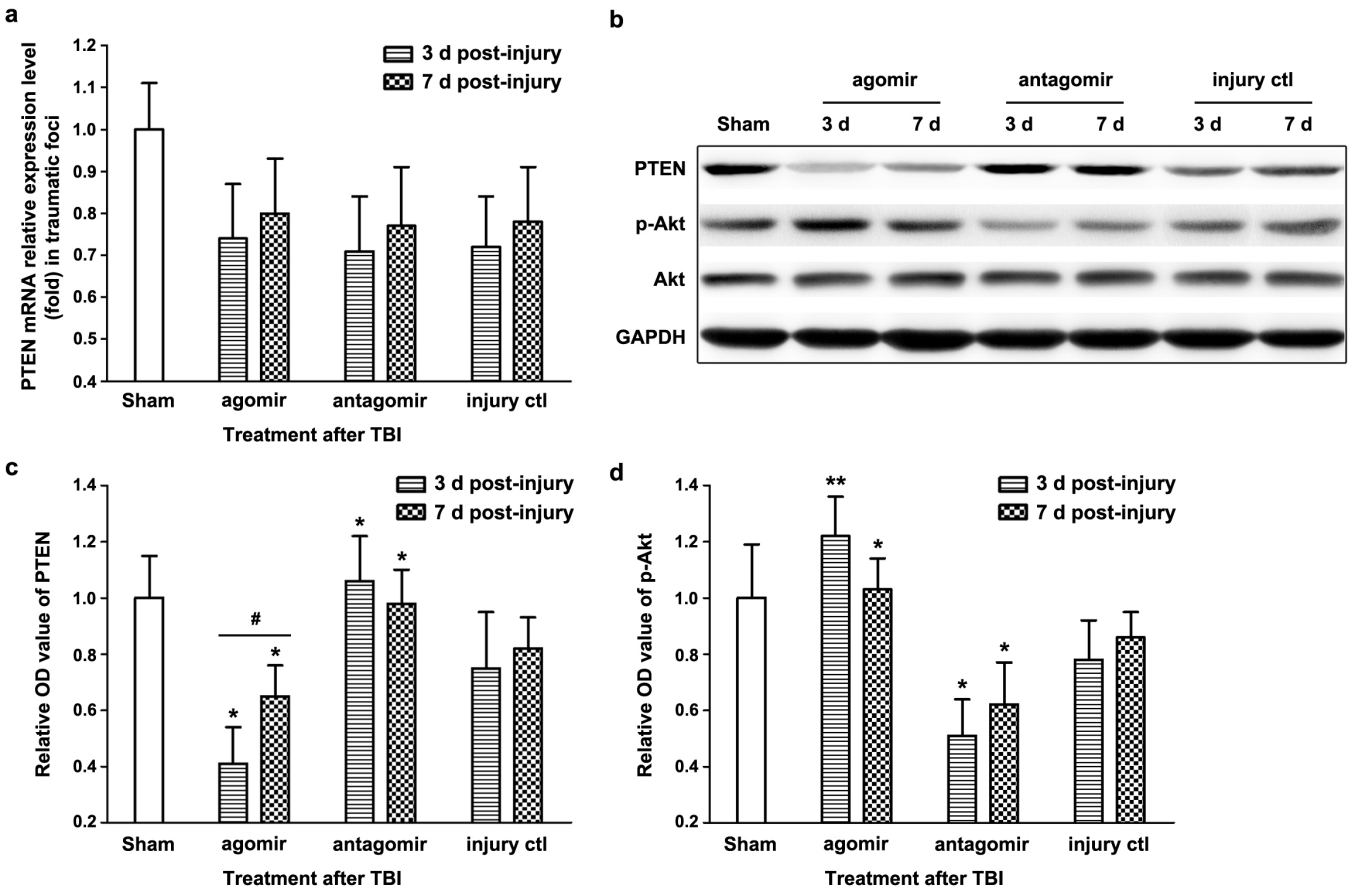
The role of Akt signaling under miR-21 regulation in the traumatic foci after TBI. The quantitative data of PTEN mRNA expression at 3 d and 7 d post-injury determined by qRT-PCR (a). The immunoblotting (b) and quantitative data (c, d) of PTEN and p-Akt at 3 d and 7 d post-injury. The data are expressed as mean ± SD. (n = 6) (*P < 0.05, **P < 0.01 versus the *injury ctl* group, ^#^P < 0.05 represent 3 d post-injury versus 7 d post-injury).

To explore the molecular mechanisms underlying the functions of miR-21 in TBI, we examined the putative target of miR-21, the PTEN/PI3K-Akt signaling pathway, at 3 d (when miR-21 inhibited apoptosis in brain) and 7d post-injury (when miR-21 promoted angiogenesis in brain). To determine whether and how miR-21 regulates PTEN expression in brain after TBI, the mRNA and protein levels of PTEN were quantified. Compared with the *injury ctl* group, no difference in the mRNA level of PTEN was observed at 3 d or 7 d post-injury (Figure 6a), while the protein level of PTEN was decreased in the *agomir* group and increased in the *antagomir* group at 3 d and 7 d post-injury (Figures 6b, 6c). Loss of PTEN can lead to Akt activation by promoting its phosphorylation at Thr308 and/or Ser473^17^. We detected Akt and phospho-Akt (p-Akt) levels in brain and found that the expression of p-Akt protein was increased in the *agomir* group and decreased in the *antagomir* group at 3 d and 7 d post-injury compared with the *injury* *ctl* group (Figure 6b, 6d). Furthermore, the Spearman's rank correlation coefficient test showed significant correlations between (1) miR-21 level and protein level of PTEN (r = −0.947, P = 0.015) and (2) miR-21 level and protein level of p-AKT (r = 0.963 P = 0.009) in the traumatic foci after TBI. Taken together, these data demonstrated that miR-21 inhibited the expression of PTEN at the post-transcriptional level and activated Akt signaling in brain after TBI.

## Discussion

Our recent studies on the expression levels of miRNAs in brain after TBI have identified increased expression of miR-21 in the cerebral cortex^14^, suggesting that miR-21 may be involved in pathological development after TBI, especially in secondary brain damage. To decipher the role of miR-21 in TBI, we employed the fluid percussion injury (FPI) rat model and manipulated (upregulated or downregulated) miR-21 level in brain by intracerebroventricular infusion of miR-21 agomir or antagomir. We detected the temporal profile of miR-21 level and MVECs – expressed miR-21 level in the traumatic foci and found that they were significantly increased (or decreased) after infusing miR-21 agomir (or antagomir) (Figure 1a, 1b). In addition, we quantified the in-situ expression of miR-21 in various cell types in the CNS including neurons, astrocytes and microglias. We found that the expression levels of miR-21 in the above cells were all manipulated by the intervention of miR-21 agomir or antagomir (Figure 1b–1e, Figure 2, Supplementary Figure 2). We expected that miR-21 oligomers would be taken up by the CNS through the cerebrospinal fluid (CSF)-contacting neural system and by MVECs through the CSF-blood substance transport. Taken together, these results suggested that intracerebroventricular infusion of miR-21 agomir or antagomir can effectively regulate the miR-21 level in the traumatic foci after TBI.

We first studied the impact of miR-21 on the neurological outcome after TBI. We found that the upregulation of miR-21 level in brain is beneficial for improving the neurological function of TBI rats, as demonstrated by an improvement in long-term neurological function, alleviation of brain edema and a decrease in lesion volume (Figure 3). Since the miR-21 level in brain was increased from 6 h to 7 d post-injury in the *injury ctl* group (Figure 1a), we believe that the elevation of miR-21 level is a protective response following TBI, and miR-21 could be a potential therapeutic target for interventions to improve the prognosis of TBI.

The secondary brain damage, which can be induced by the FPI model, includes cellular apoptosis, local blood supply impairment and other pathological changes after injury. In our previous research on TBI, we found that apoptosis can exacerbate tissue damage and lead to neurological deficiency^18^. The restoration of local vasculature in the brain can provide critical neurovascular substrates for neural remodeling and promote proliferation, migration and differentiation of neural stem cells at injured sites, therefore improving the neurological outcome^2^,^3^,^19^. In addition, our previous research on glioblastoma suggested that miR-21 inhibits apoptosis by targeting PTEN and activating Akt signaling^20^,^21^. miR-21 can also promote angiogenesis by upregulating VEGF expression through activating Akt signaling in human prostate cancer cells^22^. Based on these findings, we hypothesized that miR-21 can attenuate cellular apoptosis and promote angiogenesis in brain, and it could exert these effects while inhibiting its target gene – PTEN and activating Akt signaling.

To verify the above hypothesis, we studied the impact of miR-21 on apoptosis in brain after TBI. We found that miR-21 alleviated cellular apoptosis in the LB and the DG (Figure 4a–4c). miR-21 increased Bcl-2 expression and inhibited Bax and Caspase-3 expressions in brain (Figure 4d–4e), suggesting that miR-21 suppresses cellular apoptosis through inhibiting the mitochondrial apoptosis pathway^23^. Regarding the impact of miR-21 on angiogenesis in brain after TBI, we demonstrated that miR-21 can increase the MVD in the LB and the DG (Figure 5a–5c). Meanwhile, miR-21 promoted the expression of downstream VEGF (Figure 5d–5h), which is a fate-determining factor of the angioblastic cell lineage and an upstream inducer of the angiogenic cascade^24^. Meanwhile, the Ang-1/Tie-2 axis, which plays a crucial role in promoting angiogenesis and maintaining vascular maturation and stabilization^25^,^26^, was also activated (Figure 5d–5h). The combined treatment of VEGF and Ang-1 can promote angiogenesis in brain and is more potent than either alone as demonstrated by previous reports^27^. Consequently, miR-21 behaves as an inducer of angiogenic factors that promote angiogenesis in brain after TBI. VEGF, Ang-1 and Tie-2 are all expressed in MVECs in both physiological and pathological states. Since MVECs-expressed miR-21 was significantly upregulated in brain after TBI (Figure 1b), we inferred that the impact of miR-21 on angiogenesis was primarily regulated by the MVECs. Taken together, these findings suggested that miR-21 can inhibit apoptosis and promote angiogenesis in brain after TBI through regulating the expression of apoptosis- and angiogenesis-related molecules.

We also noted that the development of brain edema was related to the BBB leakage after TBI^28^, and activation of the Ang-1/Tie-2 axis can protect BBB stabilization^29^. Here we observed that miR-21 alleviated brain edema (Figure 1d) and promoted the expression of VEGF and the Ang-1/Tie-2 axis simultaneously after TBI (Figure 5f–5h). A similar result showing that the hyperstimulation of both VEGF and the Ang-1/Tie-2 axis in brain can promote vascular integrity and alleviate BBB leakage has been observed in research on stroke^30^,^31^. We inferred that the therapeutic effect of miR-21 on alleviating brain edema may correlate with its anti-apoptotic effect that contributes to maintaining the integrity of BBB, in addition to its impacts on vascular maturation and BBB stabilization.

Furthermore, we found that miR-21 inhibited the expression of PTEN at the post-transcriptional level and activated Akt signaling in brain after TBI (Figure 6). The PTEN/PI3K-Akt signaling pathway has been shown to play critical roles in impacting apoptosis^32^ and angiogenesis^22^ by affecting the expression of downstream molecules including Bcl-2, Bax, Caspase-3 and VEGF, which are regulated by miR-21. Therefore, the anti-apoptotic and pro-angiogenic effects of miR-21 in brain may relate to the inhibition of PTEN and the activation of Akt signaling. We also noted that both PTEN and Bcl-2^33^ are target genes for miR-21, and the Bcl-2 level was regulated (Figure 4d, 4e) with the activation of Akt signaling (Figure 6) in brain after TBI. However, we could not ascertain from this data whether the observed results are due to miR-21 targeting PTEN or Bcl-2. To clarify the mechanism, in vitro experiments including luciferase reporter assay should be performed in the future.

In conclusion, we found that miR-21 can improve the neurological outcome after TBI in rats. miR-21 inhibited apoptosis and promoted angiogenesis through regulating the expression of apoptosis- and angiogenesis-related molecules. The expression of PTEN was inhibited and Akt signaling was activated in the procedure. To further demonstrate the role of miR-21 in TBI, we are improving the transfection efficiency of miR-21 oligomers by drawing the time course of their uptake and degradation in CSF. We are evaluating toxicity of Lipofectamine to determine the optimal dose that maintains high transfection efficiency and minimal toxicity, which would provide valuable data for translational research. The mechanism underlying the role of miR-21 in regulating BBB leakage could also be further studied to determine the application prospects of miR-21 in TBI. Nevertheless, this study expands previous understanding on the functions of miR-21, suggesting that it could be a potential therapeutic target for interventions after TBI. This research opens a new avenue of therapeutic strategies for TBI by manipulating miRNA levels.

## Methods

Animal care and experiments were conducted in accordance with the Policy of Animal Care and Use Committee of Tianjin Medical University. All investigators were blinded to the treatment groups during animal surgery, data collection and analysis. Information about the antibodies used in the study are listed in Supplementary Table 1.

### Animal model of Fluid Percussion Injury (FPI)

12 month old male SD rats (n = 82, each weighing approximately 300 g) were purchased from the Chinese Academy of Military Science (Beijing, China). Briefly, rats were anesthetized with 10% chloride hydrate (3.0 mL/kg) administered by intraperitoneal injection. A 3.0 mm diameter craniotomy was drilled over the right parietal bone at 2.0 mm lateral to the sagittal suture and 3.0 mm caudal to bregma. An injury hub was created by cutting off the end of a Luer-lok and was fixed with dental cement over the craniotomy. After the injury hub was filled with sterile normal saline and connected to the FPI device (New Sun Health Products, Cedar Bluff, VA, USA), a 1.8–2.0 atm pressure was applied and monitored by an oscilloscope and an amplifier connected to the device. Any rats with a herniation of dura mater or occlusion of the injury hub were eliminated from the group. Less than 3% of rats did not reach the entry criteria into the study, and the mortality rate was approximately 5%.

### Experimental groups and intracerebroventricular infusion

Rats were randomly divided into 6 groups: miR-21-UPTM agomir (*agomir*), agomir negative control (*agomir ctl*), miR-21-DownTM antagomir (*antagomir*), antagomir negative control (*antagomir ctl*), injury control (*injury ctl*) and *sham* group. As described previously, miRNA oligomers (sequences listed in Supplementary Table 2) (GenePharma, Shanghai, China) were diluted to a final concentration of 50 μM according to the recommended concentration of 1–100 μM for intracerebroventricular infusion^34^,^35^. miRNA oligomers (5 μL) were then combined with lipofectamine-2000 (12.5 μL) (Invitrogen, Carlsbad, CA, USA) in an RNase-free PCR tube and incubated for at least 20 min at room temperature. The total amount of the 17.5 μL complexes was far below 5% of 580 μL (average cerebral spinal fluid amount of rats), so that the manipulation of intracerebroventricular infusion was unlikely to cause intracranial hypertension^36^. Intracerebroventricular infusion was administered 10 min after FPI modeling as previously reported^37^. The Hamilton brain infusion syringe was stereotaxically injected into the right lateral ventricle through a bur hole which was drilled previously (coordinates: 1.5 mm caudal to bregma; 1.1 mm lateral to midline; 4.5 mm deep from the surface of skull). Continuous infusion was maintained at the rate of 1 μL/min. The needle was withdrawn 3 min after the completion of infusion. The speed of inserting and withdrawing the needle was controlled at 1.5 mm/min. The craniotomy was closed with bone wax and the scalp was sutured. After recovery from anesthesia, rats were returned to home cage with free access to soft food and water. Rats of the *sham* group underwent the same surgical procedure without being exposed to percussion injury and fluid infusion.

### Tissue preparation

For the combined miRNA ISH and IF staining, the lesion volume quantification, the TUNEL assay, the MVD measurement and the IF staining, rats were sacrificed by transcardiac perfusion with PBS followed by 4% paraformaldehyde. The brains were then carefully removed and kept in 4% paraformaldehyde for 24 h at 4°C. After fixation, they were paraffin embedded and processed to 6 μm coronal sections. For the qRT-PCR and Western Blotting, rats were sacrificed by transcardiac perfusion with cold PBS to eliminate the RNA and protein expressed by blood cells. Brains were dissected on ice immediately. Thereafter, the traumatic foci were isolated as soon as possible. The tissues were then flash-frozen and stored in liquid nitrogen for subsequent RNA and protein extraction. Specifically, for the quantification of miR-21, VEGF, Ang-1 and Tie-2 levels in the MVECs, the microvessels were isolated from the traumatic foci by centrifugation and filtration as previously reported^38^.

### qRT-PCR

The RNA was harvested from acquired tissues using TRIzol reagent (Invitrogen, Carlsbad, CA, USA). cDNA generation and RT-PCR was performed using Hairpin-it™ miRNA/mRNA RT-PCR Quantitation kit (GenePharma, Shanghai, China). All PCR reactions were performed using standard PCR conditions. U6 served as the internal control for miR-21, and GAPDH was used as the internal control for PTEN. The cycle threshold (Ct value) was acquired using the Opticon Monitor Analysis Software (MJ Research, St. Bruno, Quebec, Canada). The data were analyzed using the 2^−ΔΔCt^ method, in which the RNA levels of the *sham* group were used as controls.

### Combined miRNA ISH and IF staining

In this test, biomarkers of neurons (MAP-2)^39^, astrocytes (GFAP)^40^ and microglias (Iba-1)^41^ were respectively counterstained with miR-21 using the miRNA ISH kit (Boster, Wuhan, China). The reagents were treated with DEPC, and containers were dried at 180°C for 8 h before use. On day 1, the paraffin sections were dewaxed, rehydrated and retrieved by heating in 10 mM citrate buffer for 20 min at 92–98°C. Next, sections underwent pretreatment with 2× standard saline citrate (SSC) for 25 min^42^. Post-fixation with 1% paraformaldehyde for 10 min and pre-hybridization with pre-hybridized solution for 2–4 h at 40°C were performed. The sections were then incubated with locked nucleic acid-modified miR-21-5p probe (sequences: 5′-TCAACATCAGTCTGATAAGCTA-3′, the locked nucleic acids were eight consecutive bases indicated by the underline) and covered with RNase-free coversclips overnight at 40°C in a slide warmer. On day 2, the sections were rinsed in progressive dilutions of SSC (2×, 0.5×, 0.2×) after hybridization. Thereafter, sections were blocked using 3% bovine serum albumin (BSA) for 30 min at 37°C and incubated overnight at 4°C with the primary antibody of the above mentioned biomarkers. On day 3, sections were blocked using ISH blocking buffer for 30 min at 37°C. They were then incubated with a mixed solution of the secondary antibody and biotinylated anti-digoxin antibody for 1 h at 37°C, followed by reacting with Avidin-cy3 for 30 min at 37°C in a dark room. The nuclei were counterstained with DAPI.

### mNSS test

The modified neurological severity score, which includes motor, sensory, reflex and balance tests, was evaluated by an observer who was blinded to the experimental conditions and treatments as previously reported^43^. The mNSS test was performed 24 h post-injury to ensure the relative uniformity of injury severity of rats in the same group. In addition, rats were examined before injury, as well as 1, 3, 7 and 14 days after TBI to study the effect of different treatments on neurological function.

### Morris Water Maze (MWM) test

The spatial acquisition trial was performed on 14–18 d post-injury, and the reference memory probe trail was conducted 24 h after the last spatial acquisition training as previously reported^44^. The entire procedure in the pool was monitored by a tracking system (Ethovision 3.0, Noldus Information Technology, Wageningen, Netherlands) to record the latency-time to reach the platform and calculate the time spent in the goal quadrant and the average swim speed.

### Brain water content measurement

The brain water content was measured using the wet-dry weight method as previously reported^45^. Briefly, animals were sacrificed at 72 h post-injury without transcardiac perfusion. The lesioned cerebral hemisphere was dissected immediately. It was weighed before and after drying in an electro-thermostatic blast oven at 80°C for 72 h. The percentage of water content was calculated using the equation: [(wet weight - dry weight)/wet weight] × 100%.

### Lesion volume quantification

We examined sections that were acquired 14 d post-injury and stained them with hematoxylin and eosin (H&E). The brain sections were then traced by the Image-pro Plus software program (Media Cybernetics, Rockvillie, MD, USA). The volume of indirect lesioned area (the intact area of the ipsilateral hemisphere is subtracted from the area of the contralateral hemisphere) was calculated, and the results were presented as a volume percentage of the lesion compared with the contralateral hemisphere as previously reported^46^.

### TUNEL assay

We examined sections that were acquired 72 h post-injury using the In Situ Cell Death Detection Kit, POD (Roche, Basel, Switzerland) as previously described^47^. Briefly, the paraffin sections were dewaxed and rehydrated, followed by incubation with protease K (20 μg/mL) for 30 min at 37°C. After being rinsed with PBS, sections were incubated with the TUNEL reaction mixture in a dark room for 2 h at 37°C. The nuclei were then counterstained with DAPI.

### Western Blotting

The SDS/PAGE and Western Blotting were performed as previously reported^48^. For densitometry, the Gene-Genius Imaging System (Syngene, Cambridge, UK) was used. Measurement of the mean pixel density of each band was performed using the ImageJ software (NIH). Measurements were standardized to GAPDH or α-tubulin loading controls.

### IF staining

The sections were dewaxed, rehydrated and retrieved by heating in 10 mM citrate buffer (pH 6.0) for 20 min at 92–98°C. They were then treated with 3% BSA and 1% normal goat serum for 30 min at 37°C to block nonspecific staining and incubated overnight at 4°C with the primary antibody. After being rinsed with PBS, the sections were incubated for 1 h at room temperature with an appropriate secondary antibody. The nuclei were counterstained with DAPI.

### MVD measurement

The MVD was determined by IF staining of CD31 (the biomarker of MVECs^49^) at 7 d post-injury as we previously reported^50^. The microvessels were defined as a cluster of immunostained CD31-positive cells with or without a lumen structure, and each cluster of CD31-positive cells were counted as one microvessel.

### Quantification for florescent staining sections

For quantitative measurement, sections were digitized under a 20× objective using a 3-CCD color video camera (Sony DXC-970MD, Japan) with an immunofluorescence microscope (Olympus IX81, Japan). They were then quantified by an investigator who was blinded to the experiment. For the TUNEL assay and IF staining, five separate slides (40 μm apart from each other) from each brain were digitized, with each slide containing three randomly selected 200× fields from the LB and the DG. The digitized images were contrast-enhanced to clearly differentiate the positively labeled cells from the background. Specifically, the immunoreactive areas of VEGF and Ang-1, which are secreted by endothelial cells, were defined as those located on the vessels (majority) and the extracellular space of endothelial cells (minority). The immunoreactive area of Tie-2 was located on the vessels. A thresholding procedure was then established to determine the proportion of immunoreactive area using the NIS-Elements BR analysis system (Nikon, Japan). For the MVD measurement, sections were digitized similar to that in the TUNEL assay, and the mean value of microvessels in the LB and the DG were respectively calculated, which were converted into the number of microvessels/mm^2^ for statistical analysis. For the combined miRNA ISH and IF staining, cells in the LB, the subdivisions of Ammon's horn (CA1 and CA3) and the DG were observed. Double immunopositive cells in five separate slides from each brain, with each slide containing three 200× fields from each of the above regions, were counted and the average of double immunopositive cells from all regions was calculated. The data were presented as the cell number in one square millimeter.

### Statistical analysis

All data are based on at least 3 independent experiments. The data are expressed as mean ± SD, except for the data from the spatial acquisition trials of the MWM test, which is expressed as mean ± SEM. Data from the spatial acquisition trial of the MWM test were analyzed using repeated measures ANOVA. For other data, statistical comparisons were analyzed using a one-way ANOVA followed by a LSD post hoc analysis or Student's t-test. A p-value of less than 0.05 was considered significant.

## Author Contributions

J.-N.Z. and C.-S.K. were responsible for experimental design. X.-T.G., P.L. and H.-C.W. developed methodology. X.-T.G., A.-L.Z., Z.-L.H., X.C. and S.-H.L. carried out the experiments. X.-T.G. and P.L. interpreted the results, performed data analysis and prepared the figures and tables. X.-T.G. wrote the manuscript. H.-C.W. reviewed and revised the manuscript. R.-C.J. provided technical support. J.-N.Z. and C.-S.K. supervised the study.

## Supplementary Material

Supplementary Information Supplementary Materials

Supplementary Information Dataset 1
