# Supplementary material for: miR-21 improves the neurological outcome after traumatic brain injury in rats
Source: Sci Rep. 2014 Oct 24;4:6718. doi: 10.1038/srep06718 (PMC4208064; doi:10.1038/srep06718)
Supplement: Supplementary Information — Supplementary Materials [file srep06718-s1.pdf]

# miR-21 improves the neurological outcome after traumatic brain injury in rats

Xin-Tong Ge[1, 2, 3], Ping Lei[2, 3, 4], Hai-Chen Wang[5] , An-Ling Zhang[2, 3, 6] , Zhao-Li Han[1, 2, 4], Xin Chen[1, 2, 3], Sheng-Hui Li[1, 2, 3], Rong-Cai Jiang[1, 2, 3], Chun-Sheng Kang[2, 3, 6], Jian-Ning Zhang[1, 2, 3, *]

[1] Laboratory of Neuro-Trauma, Tianjin Neurological Institute, Tianjin, China

[2] Key Laboratory of Post-trauma Neuro-repair and Regeneration in Central Nervous System, Ministry of Education, Tianjin, China; Tianjin Key Laboratory of Injuries, Variations and Regeneration of Nervous System, Tianjin, Tianjin, China

[3] Department of Neurosurgery, Tianjin Medical University General Hospital, Tianjin, China

[4] Department of Geriatrics, Tianjin Medical University General Hospital, Tianjin, China; Laboratory of Neuro-Trauma and Neurodegenerative Disorders, Tianjin Geriatrics Institute, Tianjin, China

[5] Department of Neurology, Duke University School of Medicine, Durham, North Carolina, U.S.A

[6] Laboratory of Neuro-Oncology, Tianjin Neurological Institute, Tianjin, China

Corresponding Author
Jian-Ning Zhang, Department of Neurosurgery, Tianjin Medical University General Hospital, Tianjin, 300052, China. Tel./Fax: +86-022-60817448; E-mail: jianningzhang@hotmail.com

**Supplementary Figure 1. Altered miR-21 level in the contralateral cerebral cortex after TBI and miR-21 oligomers intervention.**

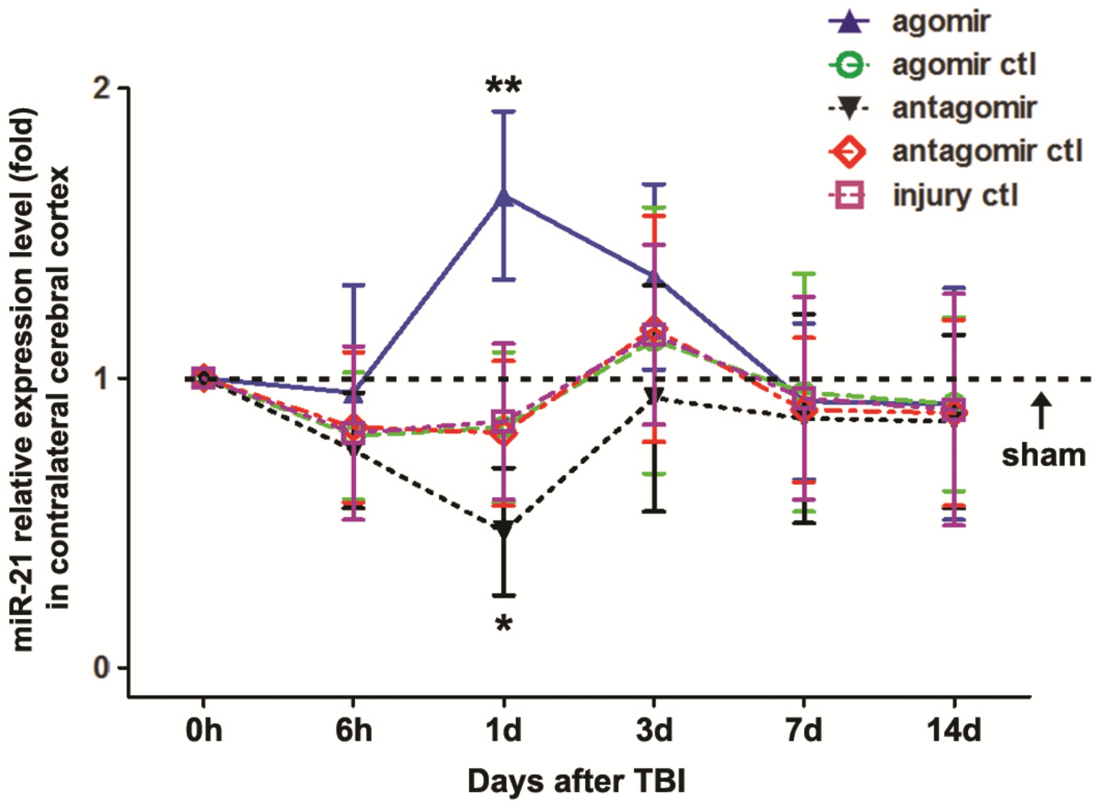


The temporal profile (from 0 h to 14 d post-injury) of miR-21 level in the contralateral cerebral cortex determined by qRT-PCR. The quantitative data were analyzed using the $2^{-\Delta\Delta Ct}$ method, in which the miR-21 levels of the *sham* group (presented as the dotted line) were used as controls. Note that the miR-21 level in the contralateral cerebral cortex were not altered after TBI (*injury ctl* group). The miR-21 level at 1 d post-injury can be upregulated (or downregulated) after infusing miR-21 agomir (or antagomir), but its variation extent is much smaller (less than 2-fold alteration) than that in the traumatic foci (more than 7-fold alteration). No difference was observed at other time-points between the *agomir* (or *antagomir*) group and *injury ctl* group. The data are expressed as mean ± SD. (n = 6) (*P<0.05, **P<0.01 versus the *injury ctl* group)

**Supplementary Figure 2. Representative images of the in-situ immunostained miR-21 in neurons, astrocytes and microglias in different areas of injured brain at 72 h post-injury compared to that of sham group.**

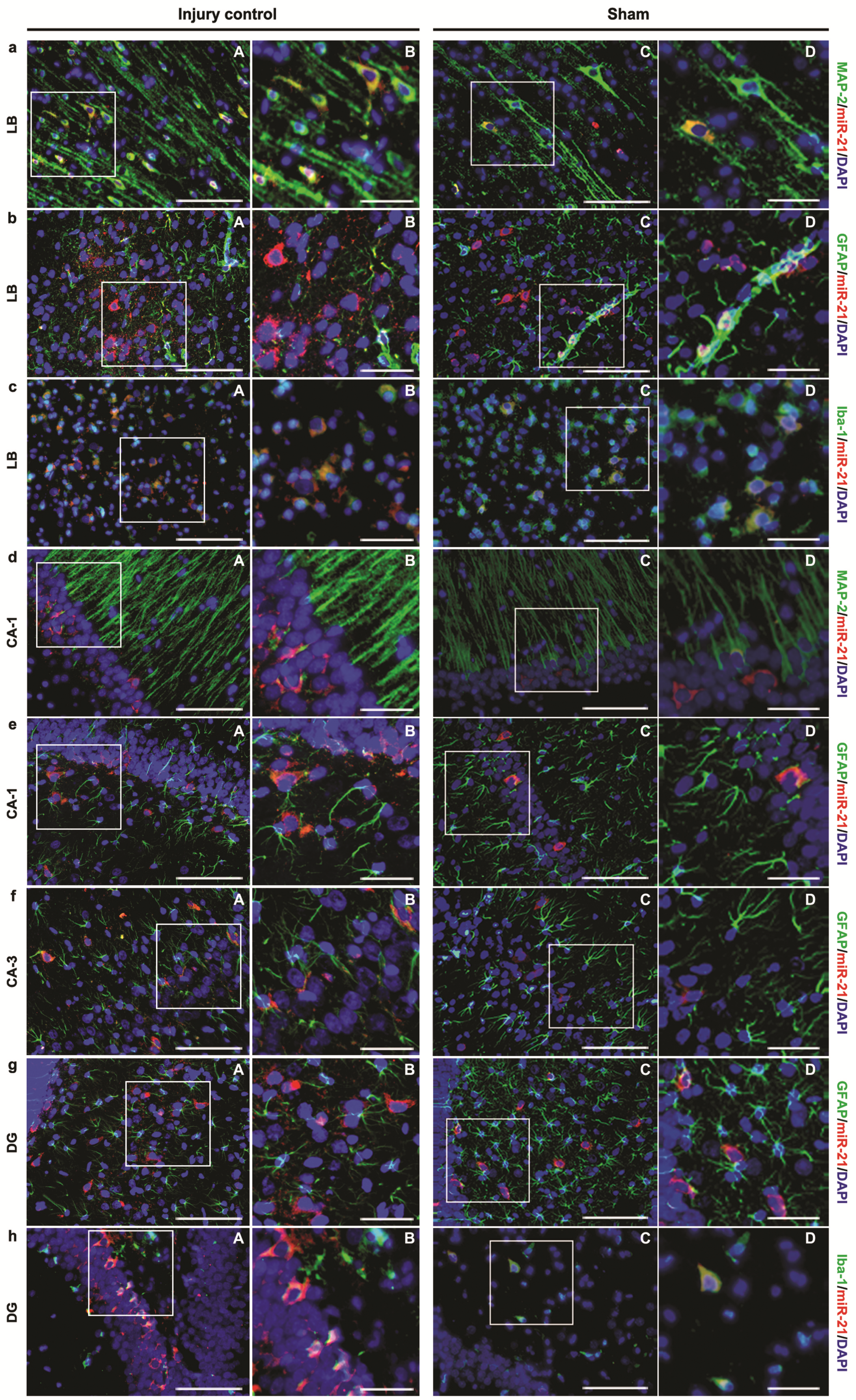


The combined staining of miR-21/MAP-2 (a), miR-21/GFAP (b), miR-21/Iba-1 (c) in the lesioned boundary of cerebral cortex (LB, scope see Figure 3e). The combined staining of miR-21/MAP-2 (d) and miR-21/GFAP (e) in the CA1 (a subdivision of Ammon's horn). The combined staining of miR-21/GFAP (f) in the CA3 (a subdivision of Ammon's horn). The combined staining of miR-21/GFAP (g) and miR-21/Iba-1 (h) in the dentate gyrus (DG). In each set of the figures, Figure A and B were captured from the *injury ctl* group, and Figure C and D were captured from the *sham* group. The immunostained areas in the white box were magnified from Figure A to B and from Figure C to D. Note that the expression of miR-21 in neurons, astrocytes and microglias were increased at 72 h post-injury. Scale bars: A, C 50μm; B, D 20μm. (n = 6)

**Supplementary Figure 3. The in-situ staining of control probe in neurons, astrocytes and microglias in brain.**

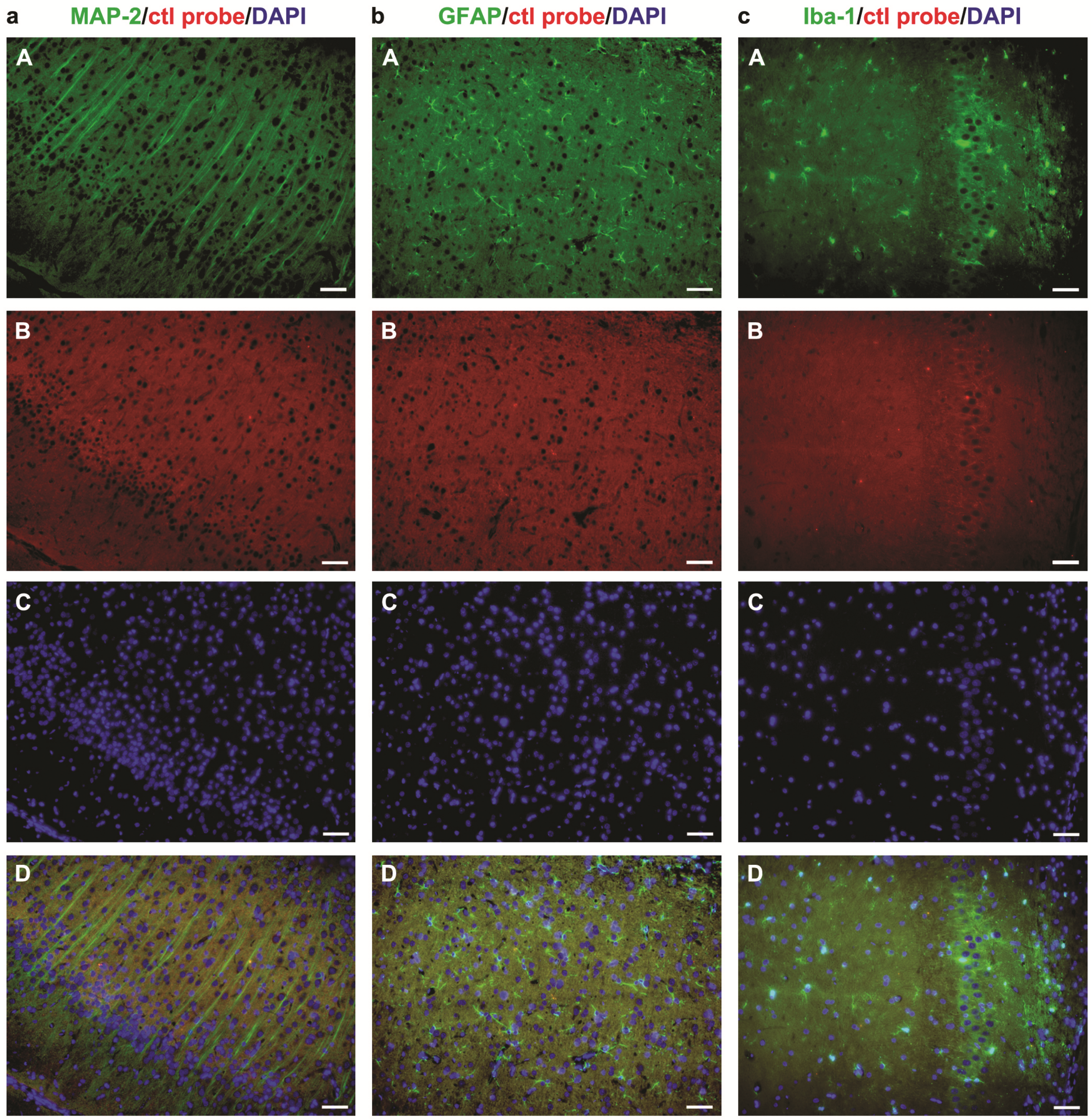


The combined staining of control probe with MAP-2 (a), GFAP (b), and Iba-1 (c) in brain. No co-labeling were observed in the sections. All figures were captured from the sham group. Scale bars: 50μm. (n = 6)

**Supplementary Figure 4. Swim speeds of each group in the Morris Water Mass test.**

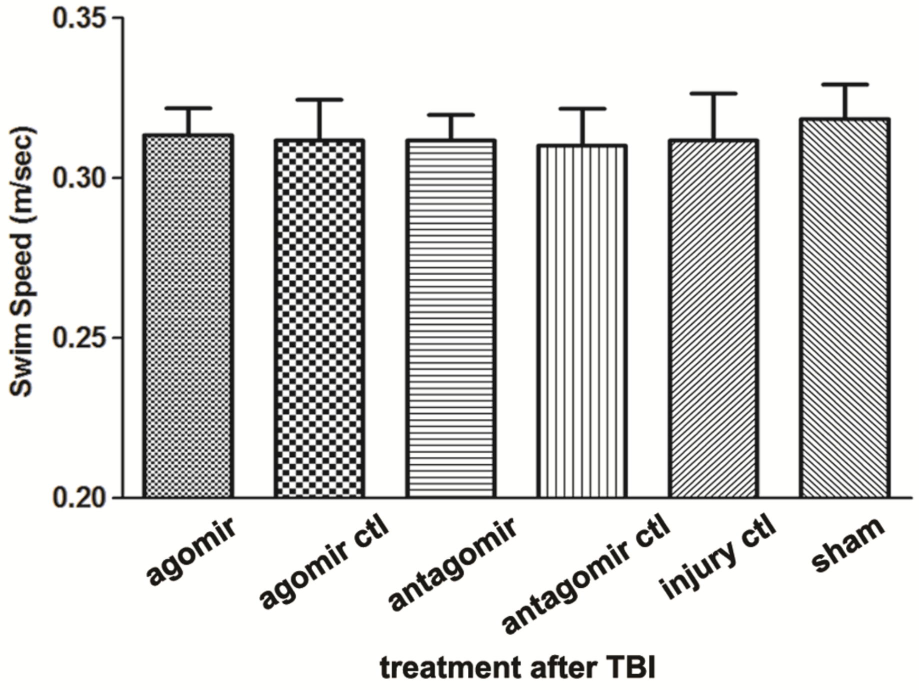


In the Morris Water Mass test, no difference was observed in swim speed among the groups (one-way ANOVA, $F(5, 30) = 0.070$, $P = 0.996$). The data are expressed as mean ± SD. ($n = 6$)

**Supplementary Figure 5. The impact of miR-21 on cellular apoptosis in contralateral brain after TBI.**

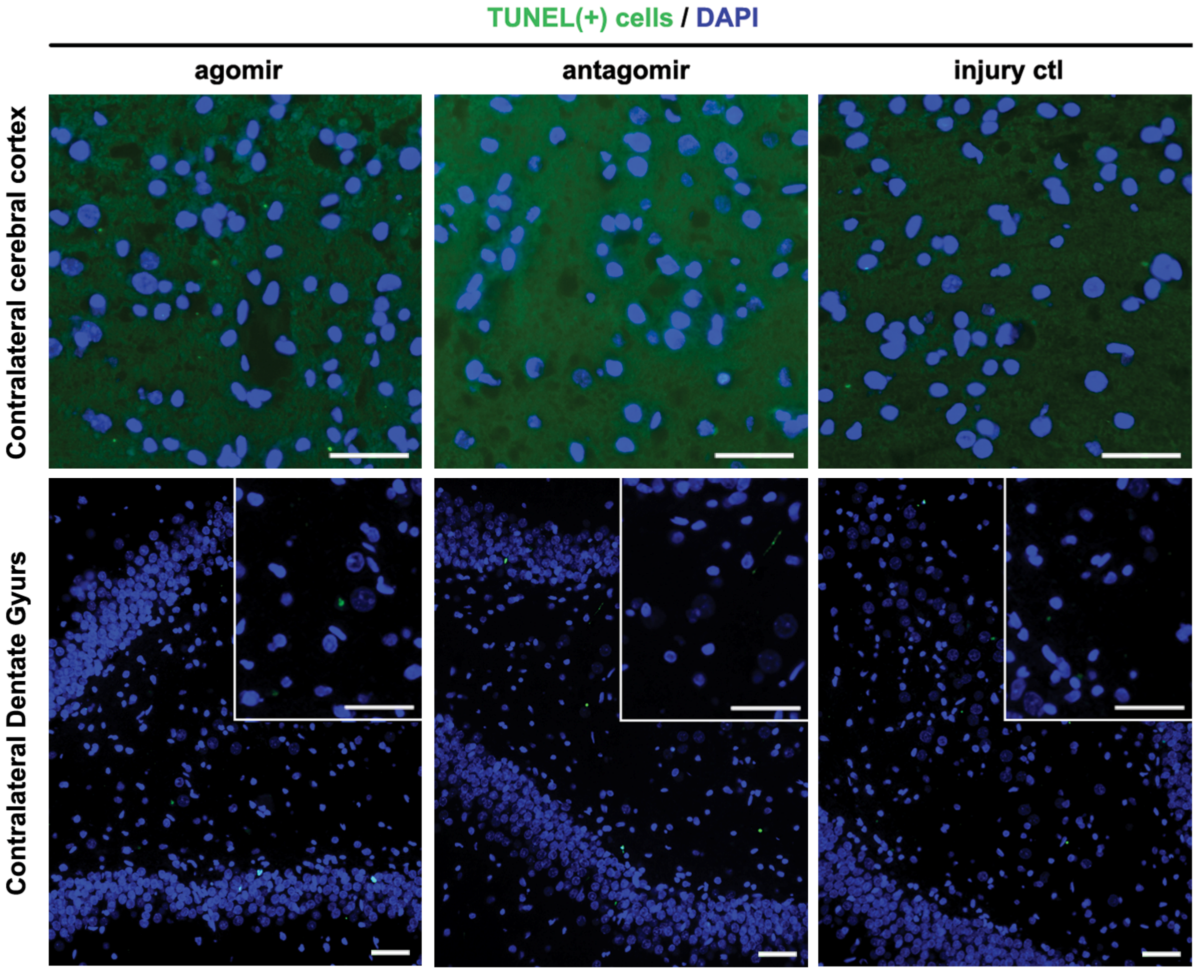


The immnuostaining of apoptotic cells in the cerebral cortex and the dentate gyrus of contralateral brain at 72 h post-injury. No difference was observed in apoptosis among the groups, suggesting that the impact of miR-21 on cellular apoptosis in brain after TBI is specific to the injury area. (n = 6)

**Supplementary Table 1. Information about the primary antibodies used in the study.**

| Antibody | Manufacturer | Catalogue | Cellular Localization | Application |
|---|---|---|---|---|
| CD31 | Santa Cruz | sc-53526 | Membrane | MVD |
| MAP-2 | Abcam | ab32454 | Cytoplasm | combined FISH and IF staining |
| GFAP | ZSJB-BIO | ZA-0117 | Cytoplasm | combined FISH and IF staining |
| Iba-1 | Abcam | ab5076 | Cytoplasm | combined FISH and IF staining |
| PTEN | CST | 9559 | Cytoplasm | WB |
| p-AKT(Ser473) | CST | 9271 | Cytoplasm | WB |
| AKT | CST | 9272 | Cytoplasm | WB |
| Bcl-2 | CST | 2876 | Mitochondrion membrane | WB |
| Bax | Abcam | ab7977 | Mitochondrion membrane | WB |
| Caspase-3 | CST | 9665 | Cytoplasm | WB |
| VEGF | Abcam | ab68334 | Secreted | IF & WB |
| Ang-1 | Abcam | ab133425 | Secreted | IF & WB |
| Tie-2 | Millipore | Ab33 05-584 | Membrane | IF & WB |
| GAPDH | ZSJB-BIO | TA-08 | Cytoplasm | WB |
| α-tubulin | Boster | BM1452 | Cytoplasm | WB |

**Supplementary Table 2. The sequences of miRNA oligomers.**

| miRNA oligomers | Sequences |
|---|---|
| miR-21 agomir | 5′-UAGCUUAUCAGACUGAUGUUGA-3′ |
| agomir negative control | 5′-UUCUCCGAACGUGUCACGUTT-3′ |
| miR-21 antagomir | 5′-UCAACAUCAGUCUGAUAAGCUA-3′ |
| antagomir negative control | 5′-CAGUACUUUUGUGUAGUACAA-3′ |
